# Arterial elasticity imaging: comparison of finite-element analysis models with high-resolution ultrasound speckle tracking

**DOI:** 10.1186/1476-7120-8-22

**Published:** 2010-06-18

**Authors:** Dae Woo Park, Michael S Richards, Jonathan M Rubin, James Hamilton, Grant H Kruger, William F Weitzel

**Affiliations:** 1 Department of Internal Medicine, University of Michigan, Ann Arbor, Michigan, USA; 2 Department of Biomedical Engineering, University of Michigan, Ann Arbor, Michigan, USA; 3 Department of Electrical and Computer Engineering, University of Rochester, Rochester, New York; 4 Department of Radiology, University of Michigan, Ann Arbor, Michigan, USA; 5 Epsilon Imaging, Inc., Ann Arbor, Michigan, USA; 6 Department of Mechanical Engineering, University of Michigan, Ann Arbor, Michigan, USA

## Abstract

**Background:**

The nonlinear mechanical properties of internal organs and tissues may be measured with unparalleled precision using ultrasound imaging with phase-sensitive speckle tracking. The many potential applications of this important noninvasive diagnostic approach include measurement of arterial stiffness, which is associated with numerous major disease processes. The accuracy of previous ultrasound measurements of arterial stiffness and vascular elasticity has been limited by the relatively low strain of nonlinear structures under normal physiologic pressure and the measurement assumption that the effect of the surrounding tissue modulus might be ignored in both physiologic and pressure equalized conditions.

**Methods:**

This study performed high-resolution ultrasound imaging of the brachial artery in a healthy adult subject under normal physiologic pressure and the use of external pressure (pressure equalization) to increase strain. These ultrasound results were compared to measurements of arterial strain as determined by finite-element analysis models with and without a surrounding tissue, which was represented by homogenous material with fixed elastic modulus.

**Results:**

Use of the pressure equalization technique during imaging resulted in average strain values of 26% and 18% at the top and sides, respectively, compared to 5% and 2%, at the top and sides, respectively, under physiologic pressure. In the artery model that included surrounding tissue, strain was 19% and 16% under pressure equalization versus 9% and 13% at the top and sides, respectively, under physiologic pressure. The model without surrounding tissue had slightly higher levels of strain under physiologic pressure compared to the other model, but the resulting strain values under pressure equalization were > 60% and did not correspond to experimental values.

**Conclusions:**

Since pressure equalization may increase the dynamic range of strain imaging, the effect of the surrounding tissue on strain should be incorporated into models of arterial strain, particularly when the pressure equalization technique is used.

## Background

Arterial stiffness is associated with numerous disease processes, including cardiovascular and renal disease, peripheral vascular occlusive disease, and diabetes. A possible cause of this increased stiffness is a change in the ratio of collagen to elastin in the extracellular matrix of the arterial media [1-3]. A variety of noninvasive techniques have been employed to measure arterial stiffness and vascular elasticity. The pulse-wave velocity (PWV) technique estimates average arterial stiffness on the basis of the travel time of a wave between two measurement sites. PWV is considered one of the best methods of measuring stiffness when time of propagation of the arterial pulse is determined between the carotid and femoral arteries [4]. But carotid-femoral PWV results may differ substantially depending on whether time is measured from the foot of the waveform (using an arterial tonometer) or the point of maximum systolic upstroke [5]. Local arterial stiffness is poorly defined by PWV and the resolution of this technique is limited by reflected waves and blood noise. Improvements in PWV estimates of local strain have been obtained by using the radiation force of ultrasound to generate propagating waves in arterial walls [6]. The same research group has distinguished between normal and calcified femoral arteries in pigs *in vivo *using vibroacoustography, which allows imaging of objects on the basis of the acoustic signal produced by two intersecting ultrasound beams [7]. Ultrasound estimates of vessel wall motion have included studies to measure femoral artery diameter and pulsatile changes in diameter to evaluate vessel thickness and stiffness in type 2 diabetes mellitus [8], carotid artery diameter and wall motion to determine the relationship of arterial calcification to vessel stiffness in end-stage renal disease [9], and femoral and carotid artery compliance in chronic dialysis patients [10]. Vessel compliance has been measured by monitoring internal pulsatile deformation in tissues surrounding the normal brachial artery [11]. Several studies have explored use of tissue Doppler imaging in pulse-wave velocity (PWV) and intraparietal strain measurements [12,13]. To maximize the accuracy of motion estimation, high-resolution ultrasound with speckle tracking algorithms have been employed [14,15] in the renal setting to measure the mechanical properties of arteries and transplant kidneys, demonstrating the potential to distinguish between normal and fibrotic tissue [16].

Blood vessels are examples of subsurface organs or tissue with highly nonlinear mechanical properties. When palpated, nonlinear structures undergo "strain hardening" where there is less strain for a given pressure differential with increasing deformation [16]. Arteries distended under normal physiologic pressure produce little strain because the normal arterial wall is a nonlinear elastic medium. This relatively low level of strain effectively limits the accuracy of measurements of the mechanical properties of arteries under physiologic conditions. However, lowering the transmural pressure on the arterial wall by applying external compression increases wall strain and deformation for a given pressure differential [16,17]. Our elasticity imaging technique achieves pressure equalization by means of continuous freehand compression or use of a blood pressure cuff. The applied external force produces internal pressure comparable to that resulting from measurement of a subject's blood pressure. The artery pulsates maximally when the applied external pressure equals the diastolic pressure, and the vessel collapses completely when the applied pressure is greater than the systolic pressure. The broader range of strain resulting from this technique may improve the ability to distinguish noninvasively between normal and diseased arterial wall if motion tracking can be performed accurately. With use of the pressure equalization procedure, ultrasound elasticity imaging with speckle tracking has potential to track motion accurately and thereby detect subtle changes in strain in the vascular wall with unprecedented precision and accuracy [16-18].

Previous ultrasound estimates of radial artery strain considered only the nonlinear elastic properties of the artery [17], noting the artery modulus to be substantially greater than that of the surrounding tissue. This allows one to approximate the modulus estimates of the artery using strain measurements from the arterial wall alone, ignoring the effects on strain of the much larger and softer surrounding tissue. However, surrounding tissue has the potential to absorb or transmit pressure to the artery and may have a particularly important effect on arterial strain when external compression is applied [16,17]. While it may seem reasonable to use only arterial wall strain measurements to approximate the modulus estimates under physiologic conditions, an interesting phenomenon occurs during pressure equalization--Not only does the artery wall modulus decrease by "unloading" the vessel, reducing transmural pressure with pressure equalization, but the opposite change occurs in the surrounding tissue. The present study evaluates the effect of the surrounding tissue modulus and validates the strain results of artery under both normal physiologic pressure and pressure equalization. Two finite-element analysis (FEA) artery models are used, one with and one without surrounding tissue modulus effects, and the FEA results are compared with *in vivo *high-resolution ultrasound data.

## Methods

### Elasticity Imaging

Local, nonlinear, high-resolution ultrasound elasticity imaging was performed on a 45-year-old healthy human male subject. The subject was enrolled for our study after providing informed consent, under a study protocol approved by our institution's Investigational Review Board. A Philips (Bothell, WA) IU22 ultrasound scanner with a 7-MHz center frequency linear array transducer was used for data collection at frame rates of approximately 180 frames per second. The subject was seated and his arm placed in the supinated position and extended forward along the sagittal plane, and resting at approximately heart level on a solid surface. The scan head was aligned on the anterior surface of the forearm so that the scan plane aligned 90° to the elbow-wrist axis (coronal plane), enabling a true accurate cross-section of the brachial artery to be obtained. Observing the B-scan images, continuous freehand positioning over the arterial region of interest was conducted, ensuring the artery remained approximately in the center of the image. Dilation of the subject's brachial artery was observed in response to the transmitted transmural pulse pressure within the artery induced by physiologic cardiac pulsations under normal atmospheric pressure. Imaging was also performed using the same method, but with the pressure equalization technique [16,17]. The external pressure was applied to the surface of the arm directly above the brachial artery using the transducer head for both tasks. When the external pressure matched the patient's diastolic blood pressure, maximal pulsation of the artery was achieved. The real-time radio-frequency (RF) data were recorded continuously for each B-mode image frame for off-line post processing.

**Figure 1:**
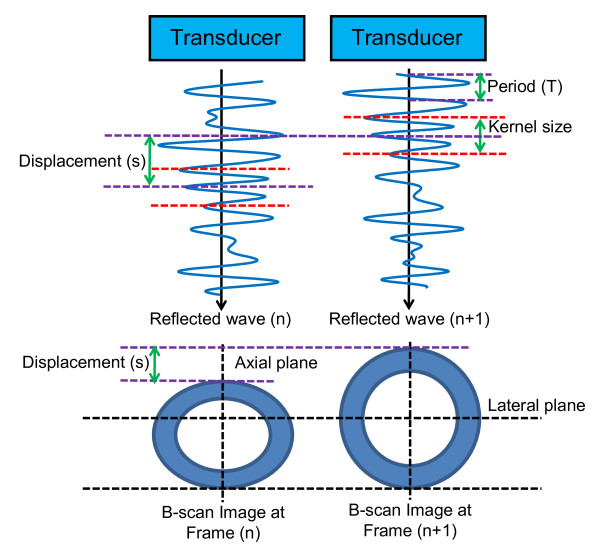
**Frame-to-frame "lag" in RF signal**. Illustration of the displacement determined from the frame-to-frame "lag" distance calculated using the correlation between the characteristic underlying radiofrequency (RF) ultrasound signal between frames.

During post-processing of the RF ultrasound signals, the displacements of the brachial artery and surrounding structures were tracked from frame-to-frame (over time), using a high-resolution, two-dimensional, correlation-based phase-sensitive speckle tracking algorithm [14,15]. Figure 1 illustrates the estimation of vessel deformation through a cross-section of an artery along the reflected post-receive beam formed RF signature. Initally, the ultrasound array is pulsed in a fashion as to create a longitudinal acoustic wave in the form of a focused beam. As the acoustic wave crosses tissue interfaces with varying acoustic impedances, a certain portion of the wave is reflected, while the rest of the energy is transmitted deeper into the tissue. The reflected signals are received by the transducer, amplified, filtered and sampled to form a sequence of discrete numeric values accurately representing the reflected waveform. Digital signal processing is then used to calculate the correlation between signals from consecutive frames. For each new reflected signal obtained, kernels (i.e., a group of sequential samples) are extracted, time shifted to various degrees, multiplied and summed with the previous frame's signals to produce a cross-correlation signal. A general form of this equation is shown in Equation 1, where x is the reflected signal, and i is the position and shift of the kernel, h, along the signal:(1)

The maximum of *y*[*i*] indicates the position of closest match between the signals. Since the reflections are due to physical structures in the tissue, mechanical deformation (i.e., compression) produces shifts in sequential reflected waveforms. The amount of signal shift corresponding to the maximum correlation represents the tissue motion. Because the transmission time of each beam is accurately controlled, the motion between time intervals and, therefore, the velocity of tissue features can be determined. The spatial (or along-the-beam signal, in the example of Fig. 1) derivative of the displacement provides the strain. For 2-D speckle tracking this process is repeated multiple times for each beam as well as between adjacent beams constituting the image. For our study the lateral and axial displacements were calculated at the position of the maximum correlation coefficient, using a correlation kernel size approximately equal to the speckle spot. The axial displacement estimate was then further refined by determining the phase zero-crossing position of the analytic signal correlation. A spatial filter twice as large as the kernel size was used to enhance signal-to-noise ratio with good spatial resolution. A weighted correlation window and spatial filtering of adjacent correlation functions were used to reduce frame-to-frame displacement error [14]. To support calculation of Lagrangian strain, interframe motion of reference frame (e.g., first frame) pixels was integrated to produce the accumulated tissue displacement. Spatial derivatives of the displacements were calculated in a region of the artery to estimate the radial normal strain. The components of strain were determined according to the direction of the ultrasound beam. Longitudinal strain is the axial strain measured along the beam direction, and lateral strain is perpendicular to the axial strain. Longitudinal strain is more accurate than lateral strain, as the maximum spatial frequency is at least an order of magnitude greater along the ultrasound beam than in the lateral (across beam) direction (see Fig. 1). Therefore, all strains were measured in the axial direction and at regions with maximum axial strain values: top, bottom and both sides of arterial wall.

### Finite-element Analysis (FEA)

For FEA modeling it is necessary to obtain accurate Young's modulus values for the materials/tissues used. A microelastometer (model 0301, ARTANN Laboratories, West Trenton, New Jersey) was used to empirically measure the strain/stress relationship on samples of bovine peripheral muscular artery and surrounding tissue obtained from a butcher shop. Cylindrical tissue specimens with a diameter of 1 mm and height of 2 mm were separately cut from the arterial wall and surrounding tissue and individually placed between the stamp and bottom plate of the microelastometer. The distance between the bottom plate and base of the microelastometer stamp was used as the reference point for displacement measurement. The tissue sample was compressed to a force of 150 g or 70% of the starting height (whichever limit is met first) to obtain the tissue's force/height dependence [19]. The stress (force per unit area) versus strain (change in length) results were calculated based on the height, force and cross-sectional area of the tissue. These quantities can be related through Equation 2 to obtain the Young's modulus of elasticity for the tissue,(2)

where *σ *and *ε *are the stress and strain, respectively, *F *is the applied force in Newtons, *L*_0 _and *A*_0 _represent the initial non-deformed length and cross-sectional area, and Δ*L *is the change in length. Because the tissue exhibits a non-linear elastic response the Young's modulus varies depending on the values of *L*_0 _and Δ*L*, with the tangent to the stress-strain curve indicating the Young's modulus for a specific *L*_0_. However, as Δ*L *→ 0 inaccuracies in measurement become more pronounced. For our analysis we assumed a linear elastic response (Hooke's Law) over the region of interest as Δ*L *is small for pulsatile arterial pressure variations considered in our research.

**Figure 2:**
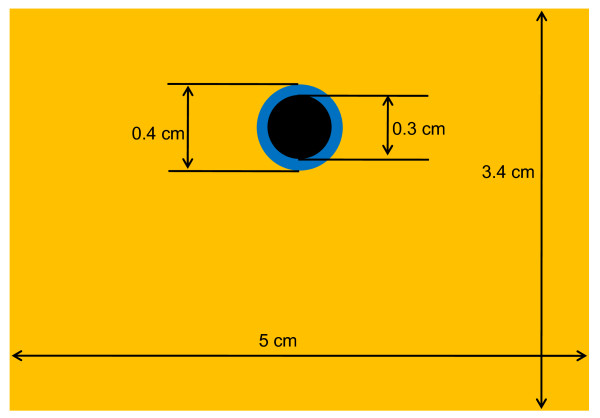
**FEA model**. Finite-element analysis (FEA) model of artery and surrounding tissue, including dimensions.

FEA of the artery models with and without surrounding tissue was performed using ABAQUS software (Simulia, Providence, Rhode Island), version 6.4, and the Young's moduli obtained in the microelastometer experiment. The axial strain was analyzed under conditions simulating both physiologic pressure and pressure equalization. A simplified model of the brachial artery and its surrounding tissue was designed (Fig. 2). For the FEA model, the quadratic-dominated element shape was used. Dimensions were determined on the basis of the ultrasound B-mode image, and boundary conditions were based on the ultrasound experiment. We assumed the volume of the surrounding tissue to be much larger than that of the artery. Thus, the boundary conditions for surrounding tissue included a fixed bottom and sides that were free to move vertically but not horizontally (horizontally symmetric conditions). Two-dimensional mesh was designed to analyze this model. Volume change of the tissue was assumed to be negligible and thus the Poisson's ratio was regarded as 0.5.

## Results

### Elasticity Imaging

**Figure 3:**
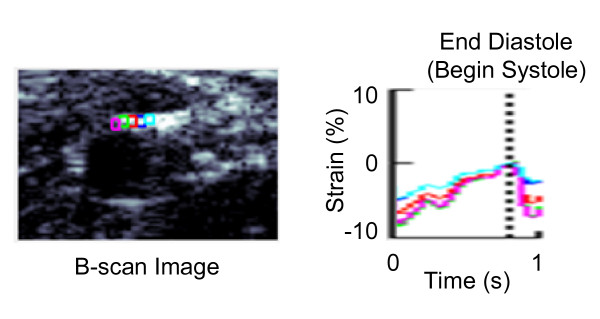
**Imaging strain vs. time plot**. B-scan image and the strain-versus-time plot during one cardiac cycle under physiologic pressure. The average strain difference can be calculated from five regions of interest along the artery wall using ultrasound elasticity imaging.

The accumulated displacement of the arterial wall was calculated with respect to the pixels of the original frame starting at diastole of the cardiac cycle. Normal accumulated strain values were obtained from the accumulated displacement, and the average strain value was estimated from five regions of interest chosen along the arterial wall during one pulsation of the artery. These strain values show axial strains, as shown in Figure 1, that were derived from spatial derivatives of the displacements in a region of the artery. The axial strain values were converted to radial normal strain by changing vector direction according to the radial normal direction of the arterial wall in order to compare the imaging and FEA strain results. Figure 3 shows the B-scan image and strain-versus-time plot of five regions of interest along the top edge of the vessel wall during one cardiac cycle under physiologic pressure. Analysis of all images showed the average strain under physiologic pressure was about -5% at the top and bottom of the arterial wall and 1% and 3% at the sides, compared to about -26% at the top and 11% and 24% at the sides under equalized pressure. (The value of average strain at the bottom of the wall under pressure equalization was disregarded as unreliable due to poor tracking). The vertical dashed line in Figure 3 represents the time at end-diastole (or onset of systole) when wall strain magnitude is at minimum.

### Finite-element Analysis (FEA)

**Figure 4:**
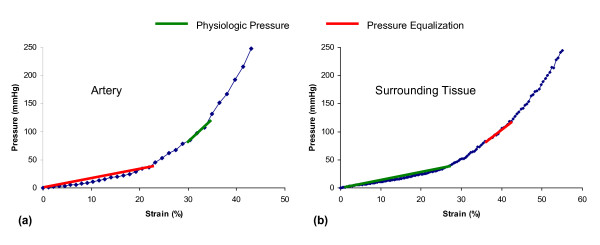
**Stress-strain curves for FEA modeling**. Stress-strain relationship for bovine arterial wall (a) and surrounding tissue (b). The linear approximations of Young's modulus used in the finite-element analysis (FEA) model are summarized in Table 1.

**Figure 5:**
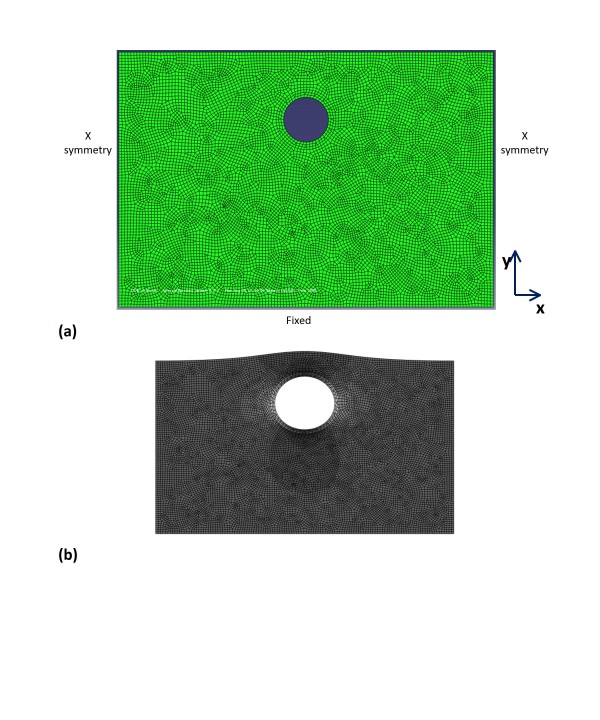
**FEA artery model**. The boundary condition (a) and strain distribution under physiologic pressure (b) of the finite-element analysis (FEA) artery model.

Figure 4 provides the results of the bovine artery and surrounding tissue microelastometer experiments as stress-strain curves. It can be seen that although strain is nonlinear overall, it can be approximated as piecewise linear function over each of the physiologic and pressure equalization ranges. Table 1 gives the Young's modulus values determined for each pressure range, using Equation 2, where σ is the change in pressure in kilopascals inside the artery and ε is the strain. Figure 5(a) shows the boundary conditions and mesh on the artery model with surrounding tissue. Figure 5(b) shows the strain distribution in the tissue under physiologic pressure. As the internal pressure increases from 80 to 120 mmHg, the radius of the artery increases, but the thickness of the arterial wall decreases. From Figure 5(b) it can be seen that the lateral sides (left and right) of the artery expand outwards, while the axial edges (top and bottom) tend to move inward (indicated as a negative strain) toward the center of the artery. In the ultrasound experiment, the subject's upper arm rests flat on a table, and pressure equalization is achieved by using the transducer to apply pressure to the arm. Thus, the bottom of the surrounding tissue is constrained while pressure is applied to the top. Under conditions of normal physiologic blood pressure of 120/80 mmHg, the transmural arterial wall pressure increases from 80 (diastolic) to 120 (systolic) mmHg. Under these conditions the artery and tissue are already under a certain amount of strain, as can be seen from Figures 4(a) and (b), resulting in a certain amount of resistance against further expansion of the vessel. A specific arterial pressure results in a force on the internal lumen wall of the artery. This force results in a displacement, or expansion of the artery. Due to the base strain offset (pre-strain) imposed due to the physiologic pressure and non-linear elastic response (steeper Young's modulus), a small displacement change occurs as the physiologic pressure pulses. As the artery expands, the arterial wall and surrounding tissue are deformed, resulting in an increasing elastic force opposing the pressure induced force. Arterial deformation reaches equilibrium when the sum of the force vectors balance (= 0), which occurs relatively quickly due to the slope of the stress-strain curve. During the pressure equalization procedure used to illicit nonlinear behavior of the arterial wall [16], an external force is applied, which results in deformation of the artery and surrounding tissue. This deformation is again balanced by the reaction force due to the elasticity of the tissue. When the reaction force exceeds the pressure-induced force, the vessel collapses. As the physiologic pressure pulses, the pressure force exceeds the external force and the artery expands again until the forces are once again in equilibrium. Due to the external force, the base strain offset (pre-strain) is removed so the expansion occurs over an area of the stress-strain curve with a lower Young's modulus, meaning that a larger displacement is necessary to balance the forces.

**Table 1:** The Young's moduli of artery and surrounding tissue under physiologic pressure and pressure equalization

	Artery	Surrounding Tissue
Physiologic pressure	118 kPa	14 kPa
Pressure equalization	22 kPa	76 kPa

**Figure 6:**
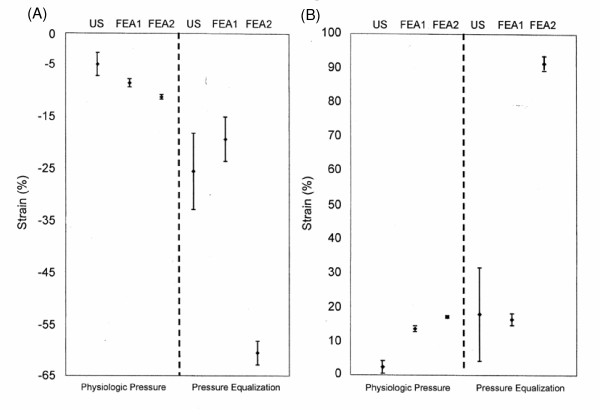
**Imaging and FEA strain values**. Average strain values and standard deviations from five regions of interest at the top (A) and sides (B) of the arterial wall under physiologic pressure and pressure equalization, as determined by high-resolution ultrasound imaging with speckle tracking (US) and finite-element analysis models with surrounding tissue (FEA1) and without surrounding tissue (FEA2).

Average strain differences and standard deviations (SDs) of n = 5 regions of interest obtained from the FEA artery models for both physiologic pressure and equalized pressure are shown in Table 2, where they are compared with the ultrasound imaging results. FEA and imaging results are also compared in Figures 6(a) and 6(b) for the regions of interest at the top and sides, respectively, of the arterial wall. Under physiologic pressure, the average strain at the top and bottom of the arterial wall in the model with surrounding tissue (FEA1) was -9%, compared to -11% in the model without surrounding tissue (FEA2). The difference in average strain values at the sides was 13% vs. 17% in the models with and without surrounding tissue, respectively. Under pressure equalization, however, the differences in average strain values between the two models were considerably greater: -20% vs. -60% in the models with and without surrounding tissue, respectively, at the top and bottom regions of interest; and 16% vs. 91% in the models with and without surrounding tissue, respectively, at the sides of the arterial wall.

**Table 2:** Average strain differences and standard deviations along the arterial wall

	Region	Ultrasound Results	FEA1	FEA2
Physiologic pressure	Top	-0.050 ± 0.023	-0.086 ± 0.008	-0.113 ± 0.004
	Bottom	-0.058 ± 0.013	-0.086 ± 0.009	-0.111 ± 0.003
	Left	0.012 ± 0.011	0.134 ± 0.011	0.170 ± 0.005
	Right	0.034 ± 0.019	0.136 ± 0.009	0.170 ± 0.004
Pressure equalization	Top	-0.256 ± 0.073	-0.194 ± 0.043	-0.606 ± 0.023
	Bottom	-0.059 ± 0.009 (Poor tracking)	-0.213 ± 0.038	-0.598 ± 0.016
	Left	0.115 ± 0.112	0.161 ± 0.021	0.914 ± 0.025
	Right	0.241 ± 0.141	0.164 ± 0.016	0.915 ± 0.019

**Figure 7:**
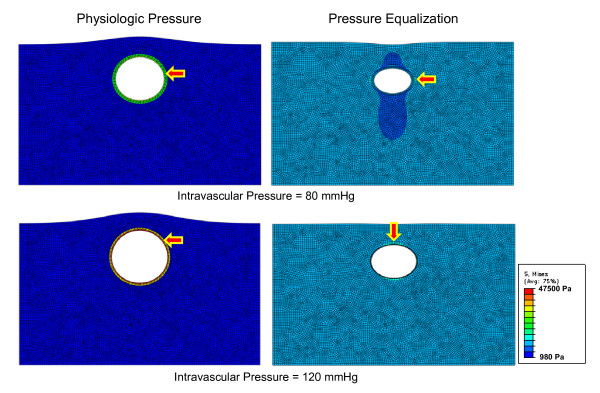
**FEA stress modeling with surrounding tissue**. The stress distribution of the artery model with surrounding tissue (FEA1) under either physiologic pressure or pressure equalization. The arrows point to high-stress regions.

Figure 7 shows the stress distribution of the artery model with surrounding tissue (FEA1) under both physiologic pressure and pressure equalization, with arrows showing high stress regions. Maximum stress increased from about 235 to 356 mmHg under physiologic pressure and was concentrated inside the arterial wall. On the other hand, the high-stress region was outside of the vessel wall under pressure equalization, when maximum stress increased from about 99 to 116 mmHg. Thus, a large portion of the external pressure was absorbed under pressure equalization, resulting in low stress on the arterial wall.

## Discussion

High-resolution ultrasound with speckle-tracking algorithms can accurately and precisely measure the motion and mechanical strain of subsurface structures and tissues such as arteries and other vessels. This noninvasive imaging technique has the clinical potential to distinguish subtle changes in arterial mechanics.

However, the arterial wall is a highly nonlinear elastic medium that undergoes little deformation when the artery is distended under normal physiologic loading. The small amount of arterial strain produced under physiologic pressure limits the range of possible measurements by elasticity imaging to characterize stiffness fully. However, previous ultrasound imaging research [16,17] has demonstrated that this limitation can be overcome by applying external pressure to lower the mean arterial pressure (MAP) that produces the low effective elastic modulus, and therefore higher radial strain, in the vessel wall. Reducing MAP decreases preload or transmural pressure and allows the arterial pulse pressure to produce much larger strain. Use of the pressure equalization technique therefore expands the dynamic range of potential strain measurements.

Previous estimates of peripheral artery strain under pressure equalization have relied on the Young's modulus of only artery [17]. We sought to investigate the effect of surrounding tissue in ultrasound elasticity measurements by comparing strain results from imaging to those of two FEA models, one employing the modulus of only artery (FEA2) and one employing the moduli of both artery and surrounding tissue (FEA1). The ultrasound and FEA strain measurements differ little under physiologic pressure. Under pressure equalization, however, the strain levels predicted by the FEA2 model are substantially greater than the levels measured by both imaging and the FEA1 model, which are relatively similar. Therefore, surrounding tissue appears to have a significant effect on arterial strain and should not be ignored in models of strain under pressure equalization. One possible hypothesis for this effect could be the relationship between the Young's moduli of the two tissues under physiologic and pressure equalization states. By evaluating Figure 4 and Table 1, it can be seen that under physiologic pressures the Young's modulus of the arterial wall is significantly greater than that of the surrounding tissue (about 8×). This means that the elasticity of the artery is predominantly responsible for balancing the expansion force produced due to the blood pressure. However, if we consider the pressure equalization state it can be seen that the Young's modulus of the surrounding tissue is approximately 3× that of the artery wall. This means that the surrounding tissue is playing a far greater role in balancing the force due to the pressure in the artery. This relationship can be clearly seen in Figure 6 (a) and (b), by comparing the FEA1 (with surrounding tissue) and FEA2 (no surrounding tissue) graphs for physiologic pressures and pressure equalization. In both cases, higher strain values are obtained when no surrounding tissue is present. Under physiologic pressures we would not e×pect to see a great difference between the FEA1 and FEA2 results. However, under pressure equalization we see much higher strain (deformation) when no surrounding tissue is present in the simulation.

A limitation of this study is the use of only one human subject for the collection of ultrasound data. The ultrasound apparatus and method of data collection were considered too experimental and impractical for use in a larger clinical investigation. The preliminary findings of the comparison of the ultrasound and FEA elasticity analyses reported here warrant further development of an ultrasound apparatus that is suitable for use in a larger clinical study.

## Conclusions

Prior studies have made important contributions to our understanding of arterial compliance. Ultrasound speckle tracking has advanced our understanding by allowing high-resolution measurements. Provocative maneuvers are being developed to increase our understanding of tissue mechanics. These results indicate the use of strain information as a diagnostic tool may need to include the effects of surrounding tissue mechanics, especially when maneuvers such as pressure equalization are used to enhance the dynamic range of elasticity imaging.

## Competing interests

The authors declare that they have no competing interests.

## Authors' contributions

DWP designed the study, performed experiments, collected and interpreted data, and drafted the manuscript. MSR, JMR, JH, and GHK interpreted data and reviewed and revised the manuscript. WFW conceived and designed the study, interpreted data, and drafted the manuscript.
